# Bis[μ-5-(5-carboxyl­ato-3-pyrid­yl)tetra­zolato-κ^3^ *N* ^1^,*N* ^5^:*N* ^2^]bis­[triaqua­zinc(II)]

**DOI:** 10.1107/S1600536809009660

**Published:** 2009-03-25

**Authors:** Haoyong Yin, Ling Wang, Qiulin Nie

**Affiliations:** a Institute of Environmental Science and Engineering, Hangzhou Dianzi University, Hangzhou 310018, People’s Republic of China; b College of Chemistry and Chemical Engineering, Xinyang Normal University, Xinyang 464000, People’s Republic of China

## Abstract

In the title complex, [Zn_2_(C_7_H_3_N_5_O_2_)_2_(H_2_O)_6_], the 5-(5-carboxyl­ato-3-pyrid­yl)tetra­zolate ligand chelates the Zn^II^ center through one pyridyl N and one tetra­zolate N atom, and uses another N atom to bridge to the second Zn atom, forming a centrosymmetric dinuclear unit. Three coordinated water mol­ecules complete the distorted octa­hedral geometry of the Zn^II^ atom. O—H⋯O and O—H⋯N hydrogen bonds involving the coordinated water mol­ecules, tetra­zolate N atoms and the carboxyl­ate group result in a three-dimensional structure.

## Related literature

For background, see: Li *et al.* (2005 ▶); Sun *et al.* (2009 ▶).
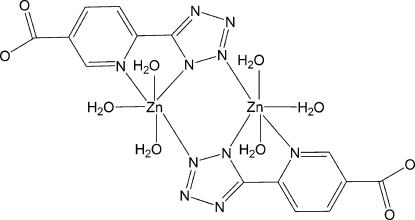


## Experimental

### 

#### Crystal data

[Zn_2_(C_7_H_3_N_5_O_2_)_2_(H_2_O)_6_]
*M* *_r_* = 617.12
Monoclinic, 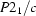

*a* = 12.751 (5) Å
*b* = 12.685 (4) Å
*c* = 6.992 (3) Å
β = 104.914 (4)°
*V* = 1092.9 (7) Å^3^
*Z* = 2
Mo *K*α radiation
μ = 2.27 mm^−1^
*T* = 295 K
0.12 × 0.08 × 0.08 mm

#### Data collection

Rigaku Mercury CCD diffractometer
Absorption correction: multi-scan (*CrystalClear*; Rigaku, 2000 ▶) *T*_min_ = 0.880, *T*_max_ = 1.000 (expected range = 0.734–0.834)
8378 measured reflections
2502 independent reflections
2146 reflections with *I* > 2σ(*I*)
*R*_int_ = 0.031

#### Refinement

*R*[*F* ^2^ > 2σ(*F* ^2^)] = 0.032
*wR*(*F* ^2^) = 0.064
*S* = 1.10
2502 reflections
187 parameters
6 restraints
H atoms treated by a mixture of independent and constrained refinement
Δρ_max_ = 0.44 e Å^−3^
Δρ_min_ = −0.31 e Å^−3^

### 

Data collection: *CrystalClear* (Rigaku, 2000 ▶); cell refinement: *CrystalClear*; data reduction: *CrystalClear*; program(s) used to solve structure: *SHELXS97* (Sheldrick, 2008 ▶); program(s) used to refine structure: *SHELXL97* (Sheldrick, 2008 ▶); molecular graphics: *X-SEED* (Barbour, 2001 ▶); software used to prepare material for publication: *SHELXL97*.

## Supplementary Material

Crystal structure: contains datablocks I, global. DOI: 10.1107/S1600536809009660/ng2558sup1.cif

Structure factors: contains datablocks I. DOI: 10.1107/S1600536809009660/ng2558Isup2.hkl

Additional supplementary materials:  crystallographic information; 3D view; checkCIF report

## Figures and Tables

**Table:** 

Zn1—O3	2.0547 (18)
Zn1—O5	2.0587 (19)
Zn1—O4	2.1317 (18)
Zn1—N5^i^	2.1333 (19)
Zn1—N2	2.1470 (18)
Zn1—N1	2.2114 (19)

**Table:** 

O3—Zn1—O5	92.12 (8)
O3—Zn1—O4	85.92 (7)
O5—Zn1—O4	177.71 (8)

Symmetry code: (i) 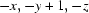.

**Table 2:** Hydrogen-bond geometry (Å, °)

*D*—H⋯*A*	*D*—H	H⋯*A*	*D*⋯*A*	*D*—H⋯*A*
O4—H4*B*⋯O2^ii^	0.846 (10)	1.942 (11)	2.782 (3)	172 (3)
O4—H4*A*⋯N3^iii^	0.841 (10)	2.110 (11)	2.940 (3)	169 (3)
O3—H3*B*⋯O1^ii^	0.849 (10)	1.875 (12)	2.712 (3)	169 (3)
O3—H3*A*⋯O1^iv^	0.846 (10)	1.880 (11)	2.719 (2)	171 (3)
O5—H5*B*⋯O1^v^	0.844 (10)	1.922 (13)	2.740 (3)	163 (3)
O5—H5*A*⋯N4^vi^	0.841 (10)	1.960 (10)	2.800 (3)	177 (3)

Symmetry codes: (ii) 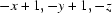; (iii) 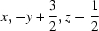; (iv) 
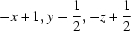
; (v) 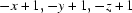; (vi) 
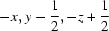
.

**Fig. 1.**
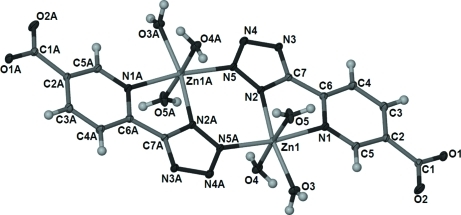
ORTEP of complex (I) with 30% thermal ellipsoids. [Symmetry code: (A) -x, 1-y, -z.]

**Fig. 2.**
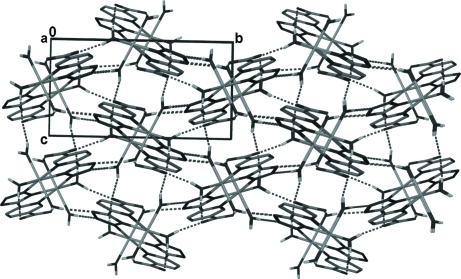
The packing structure viewed along a axis.

## supplementary crystallographic information

### Comment

Metal complexes based on tetrazol ligands have attracted great interests (Li *et al.* 2005; Sun *et al.* 2009). In the contribution, we report the title binuclear complex (I) based on tetrazol ligand obtained by *in situ* ligand synthesis.

In the structure of (I), 3-carboxylatopyridyl-6-tetrazolato ligand chelates Zn^II^ center through one pyridyl N and one tetrazolato N and another bridging tetrazolato N atom results in a centrosymmetrical binuclear unit. Three coordinated water molecules complete the distorted octahedral geometry of Zn^II^ center (Fig.1). There exist various hydrogen-bonding interactions between coordinated water molecules and tetrazol N, carboxylate group of the ligand (Table. 2). The hydrogen bonds connect binuclear complex into a three-dimensional structure (Fig.2).

### Experimental

A mixture of Zn(NO_3_)_2_.6H_2_O (149 mg, 0.5 mmol), sodium azide(33 mg, 0.5 mmol) and 6-cyanopyridine-3-carboxylic acid (74 mg, 0.5 mmol) was suspended in water (10 ml) and heated in a teflon-lined steel bomb at 160 ° C for 3 days. The colorless crystals were obtained.

### Refinement

H atoms bonded to C were located geometrically (C—H = 0.95 Å) with *U*_iso_(H) = 1.2 *U*_eq_(C). H atoms bonded to O were located by difference maps and refined with a distance restraint of O—H = 0.85 (1) Å. The displacement factors were freely refined.

### Crystal data

**Table:**

[Zn_2_(C_7_H_3_N_5_O_2_)_2_(H_2_O)_6_]	*F*(000) = 624
*M**_r_* = 617.12	*D*_x_ = 1.875 Mg m^−^^3^
Monoclinic, *P*2_1_/*c*	Mo *K*α radiation, λ = 0.71073 Å
Hall symbol: -P 2ybc	Cell parameters from 2535 reflections
*a* = 12.751 (5) Å	θ = 3.2–27.5°
*b* = 12.685 (4) Å	µ = 2.27 mm^−^^1^
*c* = 6.992 (3) Å	*T* = 295 K
β = 104.914 (4)°	Prism, colorless
*V* = 1092.9 (7) Å^3^	0.12 × 0.08 × 0.08 mm
*Z* = 2	

### Data collection

**Table:**

Rigaku Mercury CCD diffractometer	2502 independent reflections
Radiation source: fine-focus sealed tube	2146 reflections with *I* > 2σ(*I*)
graphite	*R*_int_ = 0.031
Detector resolution: 14.6306 pixels mm^-1^	θ_max_ = 27.5°, θ_min_ = 2.3°
CCD_Profile_fitting scans	*h* = −13→16
Absorption correction: multi-scan (*CrystalClear*; Rigaku, 2000)	*k* = −16→13
*T*_min_ = 0.880, *T*_max_ = 1.000	*l* = −9→8
8378 measured reflections	

### Refinement

**Table:**

Refinement on *F*^2^	Primary atom site location: structure-invariant direct methods
Least-squares matrix: full	Secondary atom site location: difference Fourier map
*R*[*F*^2^ > 2σ(*F*^2^)] = 0.032	Hydrogen site location: inferred from neighbouring sites
*wR*(*F*^2^) = 0.064	H atoms treated by a mixture of independent and constrained refinement
*S* = 1.10	*w* = 1/[σ^2^(*F*_o_^2^) + (0.0236*P*)^2^ + 0.4632*P*] where *P* = (*F*_o_^2^ + 2*F*_c_^2^)/3
2502 reflections	(Δ/σ)_max_ = 0.001
187 parameters	Δρ_max_ = 0.44 e Å^−^^3^
6 restraints	Δρ_min_ = −0.31 e Å^−^^3^

### Special details

**Table:**

Geometry. All e.s.d.'s (except the e.s.d. in the dihedral angle between two l.s. planes) are estimated using the full covariance matrix. The cell e.s.d.'s are taken into account individually in the estimation of e.s.d.'s in distances, angles and torsion angles; correlations between e.s.d.'s in cell parameters are only used when they are defined by crystal symmetry. An approximate (isotropic) treatment of cell e.s.d.'s is used for estimating e.s.d.'s involving l.s. planes.
Refinement. Refinement of *F*^2^ against ALL reflections. The weighted *R*-factor *wR* and goodness of fit *S* are based on *F*^2^, conventional *R*-factors *R* are based on *F*, with *F* set to zero for negative *F*^2^. The threshold expression of *F*^2^ > σ(*F*^2^) is used only for calculating *R*-factors(gt) *etc*. and is not relevant to the choice of reflections for refinement. *R*-factors based on *F*^2^ are statistically about twice as large as those based on *F*, and *R*- factors based on ALL data will be even larger.

### Fractional atomic coordinates and isotropic or equivalent isotropic displacement parameters (Å^2^)

**Table:**

	*x*	*y*	*z*	*U*_iso_*/*U*_eq_	
Zn1	0.16114 (2)	0.451195 (18)	0.04261 (4)	0.01891 (9)	
N1	0.29843 (15)	0.55236 (13)	0.1954 (3)	0.0199 (4)	
O2	0.62383 (14)	0.48266 (13)	0.2838 (3)	0.0345 (4)	
O1	0.67185 (13)	0.64838 (12)	0.3616 (2)	0.0273 (4)	
N2	0.08352 (15)	0.59050 (14)	0.1144 (3)	0.0184 (4)	
C1	0.60194 (19)	0.57469 (18)	0.3163 (3)	0.0219 (5)	
C4	0.3458 (2)	0.72742 (18)	0.3169 (4)	0.0258 (5)	
H4	0.3239	0.7953	0.3491	0.031*	
C6	0.26966 (19)	0.65036 (16)	0.2412 (3)	0.0196 (5)	
C3	0.4544 (2)	0.70372 (18)	0.3447 (4)	0.0260 (5)	
H3	0.5079	0.7557	0.3958	0.031*	
C2	0.48558 (18)	0.60427 (17)	0.2980 (3)	0.0193 (5)	
C5	0.40336 (19)	0.53119 (17)	0.2249 (3)	0.0217 (5)	
H5	0.4236	0.4624	0.1944	0.026*	
O3	0.26729 (15)	0.34205 (13)	−0.0178 (3)	0.0278 (4)	
O4	0.16780 (16)	0.52386 (13)	−0.2289 (3)	0.0277 (4)	
C7	0.15272 (19)	0.66661 (16)	0.1971 (3)	0.0184 (5)	
O5	0.16098 (16)	0.37901 (15)	0.3062 (3)	0.0353 (5)	
N5	−0.01525 (15)	0.63182 (14)	0.0916 (3)	0.0195 (4)	
N4	−0.00558 (16)	0.72963 (15)	0.1574 (3)	0.0250 (4)	
N3	0.09973 (16)	0.75412 (15)	0.2242 (3)	0.0245 (4)	
H4A	0.141 (2)	0.5845 (12)	−0.254 (4)	0.041 (9)*	
H3A	0.289 (2)	0.2851 (13)	0.042 (4)	0.040 (8)*	
H4B	0.2293 (14)	0.527 (2)	−0.254 (5)	0.047 (9)*	
H3B	0.279 (2)	0.339 (2)	−0.132 (2)	0.045 (9)*	
H5A	0.114 (2)	0.336 (2)	0.320 (5)	0.058 (10)*	
H5B	0.2086 (18)	0.383 (2)	0.415 (2)	0.040 (8)*	

### Atomic displacement parameters (Å^2^)

**Table:**

	*U*^11^	*U*^22^	*U*^33^	*U*^12^	*U*^13^	*U*^23^
Zn1	0.01593 (16)	0.01373 (14)	0.02694 (15)	−0.00018 (10)	0.00528 (11)	−0.00126 (10)
N1	0.0160 (10)	0.0159 (9)	0.0274 (10)	−0.0006 (7)	0.0051 (8)	−0.0027 (8)
O2	0.0210 (10)	0.0243 (9)	0.0606 (12)	0.0007 (7)	0.0146 (9)	−0.0065 (8)
O1	0.0170 (9)	0.0271 (9)	0.0372 (10)	−0.0048 (7)	0.0059 (8)	−0.0073 (7)
N2	0.0140 (10)	0.0172 (9)	0.0240 (10)	0.0005 (7)	0.0051 (8)	−0.0017 (7)
C1	0.0166 (13)	0.0276 (13)	0.0222 (12)	−0.0001 (9)	0.0061 (10)	−0.0008 (9)
C4	0.0196 (13)	0.0194 (11)	0.0382 (14)	0.0004 (9)	0.0071 (11)	−0.0072 (10)
C6	0.0188 (13)	0.0170 (11)	0.0228 (11)	0.0007 (9)	0.0051 (10)	−0.0022 (9)
C3	0.0206 (13)	0.0215 (12)	0.0344 (14)	−0.0052 (10)	0.0042 (11)	−0.0072 (10)
C2	0.0146 (12)	0.0207 (11)	0.0222 (12)	−0.0003 (9)	0.0039 (9)	−0.0007 (9)
C5	0.0173 (13)	0.0192 (11)	0.0288 (12)	0.0011 (9)	0.0063 (10)	−0.0016 (9)
O3	0.0332 (11)	0.0185 (9)	0.0347 (10)	0.0079 (7)	0.0141 (9)	0.0029 (8)
O4	0.0258 (11)	0.0238 (9)	0.0367 (10)	0.0053 (7)	0.0139 (9)	0.0075 (8)
C7	0.0168 (12)	0.0167 (10)	0.0218 (11)	−0.0013 (8)	0.0050 (9)	−0.0036 (9)
O5	0.0301 (12)	0.0394 (11)	0.0302 (10)	−0.0183 (9)	−0.0033 (9)	0.0129 (8)
N5	0.0135 (10)	0.0175 (9)	0.0273 (10)	0.0009 (7)	0.0050 (8)	−0.0028 (8)
N4	0.0185 (11)	0.0199 (10)	0.0363 (12)	0.0006 (8)	0.0067 (9)	−0.0077 (8)
N3	0.0170 (11)	0.0202 (10)	0.0353 (11)	0.0011 (8)	0.0048 (9)	−0.0089 (8)

### Geometric parameters (Å, °)

**Table:**

Zn1—O3	2.0547 (18)	C6—C7	1.457 (3)
Zn1—O5	2.0587 (19)	C3—C2	1.386 (3)
Zn1—O4	2.1317 (18)	C3—H3	0.9500
Zn1—N5^i^	2.1333 (19)	C2—C5	1.394 (3)
Zn1—N2	2.1470 (18)	C5—H5	0.9500
Zn1—N1	2.2114 (19)	O3—H3A	0.846 (10)
N1—C5	1.328 (3)	O3—H3B	0.849 (10)
N1—C6	1.358 (3)	O4—H4A	0.841 (10)
O2—C1	1.235 (3)	O4—H4B	0.846 (10)
O1—C1	1.274 (3)	C7—N3	1.338 (3)
N2—C7	1.335 (3)	O5—H5A	0.841 (10)
N2—N5	1.335 (2)	O5—H5B	0.844 (10)
C1—C2	1.504 (3)	N5—N4	1.318 (3)
C4—C3	1.381 (3)	N5—Zn1^i^	2.1333 (19)
C4—C6	1.384 (3)	N4—N3	1.340 (3)
C4—H4	0.9500		
			
O3—Zn1—O5	92.12 (8)	N1—C6—C7	113.79 (19)
O3—Zn1—O4	85.92 (7)	C4—C6—C7	124.1 (2)
O5—Zn1—O4	177.71 (8)	C4—C3—C2	120.2 (2)
O3—Zn1—N5^i^	97.06 (7)	C4—C3—H3	119.9
O5—Zn1—N5^i^	88.43 (7)	C2—C3—H3	119.9
O4—Zn1—N5^i^	92.97 (7)	C3—C2—C5	117.2 (2)
O3—Zn1—N2	166.01 (7)	C3—C2—C1	122.9 (2)
O5—Zn1—N2	92.86 (8)	C5—C2—C1	119.8 (2)
O4—Zn1—N2	88.79 (7)	N1—C5—C2	123.7 (2)
N5^i^—Zn1—N2	96.13 (7)	N1—C5—H5	118.1
O3—Zn1—N1	90.51 (7)	C2—C5—H5	118.1
O5—Zn1—N1	90.49 (7)	Zn1—O3—H3A	128.6 (19)
O4—Zn1—N1	88.35 (7)	Zn1—O3—H3B	121 (2)
N5^i^—Zn1—N1	172.39 (7)	H3A—O3—H3B	108 (3)
N2—Zn1—N1	76.39 (7)	Zn1—O4—H4A	118.2 (19)
C5—N1—C6	118.12 (19)	Zn1—O4—H4B	117 (2)
C5—N1—Zn1	126.76 (14)	H4A—O4—H4B	105 (3)
C6—N1—Zn1	114.64 (15)	N2—C7—N3	111.1 (2)
C7—N2—N5	105.42 (17)	N2—C7—C6	121.04 (19)
C7—N2—Zn1	113.79 (15)	N3—C7—C6	127.8 (2)
N5—N2—Zn1	140.58 (14)	Zn1—O5—H5A	124 (2)
O2—C1—O1	124.2 (2)	Zn1—O5—H5B	128 (2)
O2—C1—C2	119.0 (2)	H5A—O5—H5B	108 (3)
O1—C1—C2	116.8 (2)	N4—N5—N2	109.10 (17)
C3—C4—C6	118.7 (2)	N4—N5—Zn1^i^	127.65 (14)
C3—C4—H4	120.7	N2—N5—Zn1^i^	123.21 (13)
C6—C4—H4	120.7	N5—N4—N3	109.59 (17)
N1—C6—C4	122.1 (2)	C7—N3—N4	104.80 (18)

Symmetry codes: (i) −*x*, −*y*+1, −*z*.

### Hydrogen-bond geometry (Å, °)

**Table:**

*D*—H···*A*	*D*—H	H···*A*	*D*···*A*	*D*—H···*A*
O4—H4B···O2^ii^	0.85 (1)	1.94 (1)	2.782 (3)	172 (3)
O4—H4A···N3^iii^	0.84 (1)	2.11 (1)	2.940 (3)	169 (3)
O3—H3B···O1^ii^	0.85 (1)	1.88 (1)	2.712 (3)	169 (3)
O3—H3A···O1^iv^	0.85 (1)	1.88 (1)	2.719 (2)	171 (3)
O5—H5B···O1^v^	0.84 (1)	1.92 (1)	2.740 (3)	163 (3)
O5—H5A···N4^vi^	0.84 (1)	1.96 (1)	2.800 (3)	177 (3)

Symmetry codes: (ii) −*x*+1, −*y*+1, −*z*; (iii) *x*, −*y*+3/2, *z*−1/2; (iv) −*x*+1, *y*−1/2, −*z*+1/2; (v) −*x*+1, −*y*+1, −*z*+1; (vi) −*x*, *y*−1/2, −*z*+1/2.
